# Vitamin D and coronavirus disease 2019 (COVID-19): rapid evidence review

**DOI:** 10.1007/s40520-021-01894-z

**Published:** 2021-06-12

**Authors:** Zahra Raisi-Estabragh, Adrian R. Martineau, Elizabeth M. Curtis, Rebecca J. Moon, Andrea Darling, Susan Lanham-New, Kate A. Ward, Cyrus Cooper, Patricia B. Munroe, Steffen E. Petersen, Nicholas C. Harvey

**Affiliations:** 1 William Harvey Research Institute, NIHR Barts Biomedical Research Centre, Queen Mary University of London, London, UK; 2 Barts Heart Centre, St Bartholomew’s Hospital, Barts Health NHS Trust, London, UK; 3 Institute of Population Health Sciences, Barts and The London School of Medicine and Dentistry, Queen Mary University of London, London, UK; 4 MRC Lifecourse Epidemiology Unit, University of Southampton, Southampton General Hospital, Southampton, SO16 6YD UK; 5 Nutrition, Food and Exercise Sciences Department, School of Biosciences and Medicine, Faculty of Health and Medical Sciences, University of Surrey, Guildford, UK; 6 NIHR Southampton Biomedical Research Centre, University of Southampton and University Hospital Southampton NHS Foundation Trust, Southampton, UK; 7 NIHR Oxford Biomedical Research Centre, University of Oxford, Oxford, UK

**Keywords:** Severe acute respiratory syndrome coronavirus-2 (SARS-CoV-2), Coronavirus disease 2019 (COVID-19), Respiratory infection, Vitamin D, Vitamin D deficiency, Osteoporosis, Musculoskeletal health

## Abstract

**Background:**

The rapid global spread of severe acute respiratory syndrome coronavirus 2 (SARS-CoV-2), the virus that causes coronavirus disease 2019 (COVID-19), has re-ignited interest in the possible role of vitamin D in modulation of host responses to respiratory pathogens. Indeed, vitamin D supplementation has been proposed as a potential preventative or therapeutic strategy. Recommendations for any intervention, particularly in the context of a potentially fatal pandemic infection, should be strictly based on clinically informed appraisal of the evidence base. In this narrative review, we examine current evidence relating to vitamin D and COVID-19 and consider the most appropriate practical recommendations.

**Observations:**

Although there are a growing number of studies investigating the links between vitamin D and COVID-19, they are mostly small and observational with high risk of bias, residual confounding, and reverse causality. Extrapolation of molecular actions of 1,25(OH)_2_-vitamin D to an effect of increased 25(OH)-vitamin D as a result of vitamin D supplementation is generally unfounded, as is the automatic conclusion of causal mechanisms from observational studies linking low 25(OH)-vitamin D to incident disease. Efficacy is ideally demonstrated in the context of adequately powered randomised intervention studies, although such approaches may not always be feasible.

**Conclusions:**

At present, evidence to support vitamin D supplementation for the prevention or treatment of COVID-19 is inconclusive. In the absence of any further compelling data, adherence to existing national guidance on vitamin D supplementation to prevent vitamin D deficiency, predicated principally on maintaining musculoskeletal health, appears appropriate.

## Introduction

The importance of vitamin D for regulation of calcium and phosphate balance in musculoskeletal physiology is well established [1]. Vitamin D receptors are also expressed by many non-skeletal tissues suggesting a broader role for vitamin D in human health, particularly in modulating immune system activities [2–7]. Many immune cells have been found to express vitamin D receptors, including T and B cells, monocytes, macrophages, and dendritic cells [8]. It has been proposed that vitamin D may augment the first line of defence against invading pathogens and suppress the adaptive immune response, mitigating autoimmune conditions [2]. Indeed, a growing body of evidence suggests multiple biological roles for vitamin D. However, while many observational studies have demonstrated associations between low 25(OH)-vitamin D [25(OH)D] levels and a wide range of non-musculoskeletal morbidities and increased mortality, results from randomised controlled trials (RCTs) of vitamin D supplementation in these contexts have been less consistent [9, 10].

The rapid global spread of coronavirus disease 2019 (COVID-19), which is caused by severe acute respiratory syndrome coronavirus 2 (SARS-CoV-2) infection, has renewed interest in the possible role of vitamin D in modulating the immune response to respiratory infections. Indeed, widespread vitamin D supplementation has been proposed as a preventative health measure [11]. However, at present the evidence-base is of insufficient quality to support such recommendations.

Importantly, while the active 1,25(OH)_2_-vitamin D form has been shown to have a role within the immune system [12], evidence linking low 25(OH)D levels (the main circulating form, which correlates poorly to 1,25(OH)_2_-vitamin D) with increased risk or severity of COVID-19 has been inconsistent [13]. The non-COVID-19 literature contains numerous examples of 25(OH)D-morbidity associations which could be attributable to confounding or reverse causation [14]. An example, demonstrated using Mendelian randomisation, is of the observational association between low 25(OH)D levels and obesity, which in causal analyses implicate obesity as driving lower 25(OH)D rather than the other way round [15]. Furthermore, 25(OH)D levels are determined by sunlight exposure to the skin, supplement use, diet, and comorbidity (since, in some clinical contexts, 25(OH)D may be a negative acute phase reactant [16–19]). As such, vitamin D status is closely linked to general health and the potential for confounding in observational studies is high [14].

While vitamin D (as cholecalciferol [vitamin D_3_] or ergocalciferol [vitamin D_2_]) has a wide therapeutic window with a relatively low risk of toxicity, the easy availability of high-dose supplements (particularly on the internet) may increase the likelihood of such rare events in the population [20]. A further consideration in the context of the current pandemic is the potential for adverse health behaviour resulting from a perception of protection gained from taking supplements. Thus, it is important that a strictly evidence-based approach, together with health economic assessment (which is beyond the scope of this review), underpin any health recommendations in relation to the use of vitamin D supplements to prevent or treat COVID-19. In this paper, we review the pertinent literature relating to vitamin D and respiratory infections, with a particular focus on COVID-19.

## Search strategy

We searched Ovid Medline electronic database (1946 to May 14, 2021) using the following Medical Subject Heading (MeSH) terms combined using Boolean operators: [(“COVID-19” OR “SARS-CoV-2” OR “Severe Acute Respiratory Syndrome”) AND (“Vitamin D” OR “Vitamin D deficiency”)]. To ensure wide breadth of the search, we exploded the MeSH terms and included all subheadings. Papers were selected for inclusion in the review based on title and abstract screening followed by full text review, if appropriate. Further relevant studies were identified through cross referencing and author searches. Ongoing and planned studies were identified from searching https://clinicaltrials.gov. With regards summarising evidence relating to vitamin D and non-COVID-19 acute respiratory infections, we selected the latest and most comprehensive systematic evidence reviews on the topic.

### Vitamin D and acute respiratory infections

The role of vitamin D in acute respiratory infections has been examined in multiple studies, including a number of clinical trials. A recent meta-analysis of stratified aggregate data from 48,488 participants in 43 RCTs testing the effect of vitamin D on the risk of incident acute respiratory infections in adults and children demonstrated a modest protective effect from vitamin D supplementation (OR 0.92, 95% CI 0.86 to 0.99) [21]. There was significant variation in baseline 25(OH)D status of participants and in supplementation regimens. In many of the included studies, the diagnosis of acute respiratory infection was often ascertained from self-report. Overall, there was high between-study heterogeneity and the possibility of publication bias towards studies reporting a protective effect of supplementation. A second meta-analysis found that vitamin D supplementation did not reduce the risk of acute respiratory infections in 15 RCTs including healthy adults and children [22]. Again, there was substantial heterogeneity in the results, which might partly reflect differential effects of the intervention in different populations or differences in study designs. Overall, while the totality of the evidence base suggests the potential for a protective effect of vitamin D on risk of acute respiratory infections, there is inconsistency across studies and in particular definitive evidence in the older populations, who are most at risk from COVID-19, is still lacking.

### Vitamin D and COVID-19

#### In vitro studies

25(OH)D is the pre-hormone to the biologically active 1,25(OH)_2_-vitamin D, which has recognized in vitro immunomodulatory activity [8]. Mok et al. [23] sought to investigate the role of calcitriol in the context of COVID-19 using animal (monkey) and human (hepatoma, nasal epithelial) cell lines. The authors demonstrate potent activity of calcitriol against SARS-CoV-2 at a cellular level, but whether these finding can be translated into an effect of vitamin D supplementation at daily/bolus doses remains to be studied [23]. Overall, the in vitro data suggest that the vitamin D pathway may be a biologically plausible target in prevention or treatment of COVID-19. This hypothesis has prompted many researchers to examine the relationship between 25(OH)D and COVID-19 outcomes in ecological, observational, and interventional studies.

#### Ecological studies

In several early reports, researchers noted that countries with lower national average 25(OH)D levels had higher COVID-19 cases per head of population. For instance, Ilie et al. [24] reported a significant negative correlation of average population serum 25(OH)D levels with the number of COVID-19 cases and deaths per million population across 20 European countries. A similar study focused specifically on older individuals across 12 European countries, also reported an un-adjusted association between lower average population 25(OH)D and greater COVID-19 cases per million population [25]. In another study, Walrand et al. [26] estimated the date of sudden surge of COVID-19 cases (surge date) for 18 European countries using a model fitted based on daily new cases reported over the previous 2 months in Autumn 2020. They reported correlation of country surge date with latitude; specifically, they linked the surge date to when a country’s sun-derived UV daily dose dropped to below 34% of that of 0º latitude. Furthermore, the authors demonstrated that this might be explained by seasonal drop in 25(OH)D, as estimated using published data on population seasonal variations in vitamin D. In a later study, Singh et al. [27], hypothesised that, given a true protective effect of vitamin D, correlation between vitamin D levels and COVID-19 outcomes should be strengthened after the infection peak. To investigate, they estimate the association of average national vitamin D levels (obtained from previously published reports) and publicly available data on COVID-19 cases and deaths in 20 European countries after the first infection peak of COVID-19 [27]. While the authors document a significant negative correlation between vitamin D levels and COVID-19 cases per million population, the association between vitamin D and COVID-19 death was not statistically significant. It is well recognised that such ecological analyses are highly prone to confounding and may not delineate causal associations [28]. Other factors such as population demographics, density of housing, obesity, lifestyle and dietary factors, together with the potential for underlying genetic differences and inconsistency between different assays [29], may all influence such associations.

#### Observational studies

Several studies in patient cohorts have compared serum 25(OH)D levels between individuals with and without COVID-19. D’Avolio et al. [30] compared serum 25(OH)D (measured within 7 weeks prior to testing) in patients with a positive COVID-19 test (*n* = 27) with those with symptoms of respiratory infection and a negative test (*n* = 80) and with a historic pre-pandemic cohort (*n* = 1377). They report significantly lower 25(OH)D levels in the test positive cohort compared to the test negative and the historic cohorts. There was no significant difference between 25(OH)D levels of the test negative and historic groups. In a study of individuals with Parkinson’s disease, Fasano et al. [31] report that individuals without COVID-19 were more likely to take vitamin D supplementation compared to those with probable or confirmed COVID-19. These unadjusted associations suggest a potential link between low vitamin D status and increased susceptibility to COVID-19; however, observed associations may be influenced by confounding factors.

Kaufman et al. [32] present a large retrospective observational study using data from a national clinical laboratory database in the Unites States (US). SARS-CoV-2 test results for 191,779 patients were matched to serum 25(OH)D results recorded in the preceding 12 months. Geographic latitude and ethnicity were estimated by matching residential ZIP codes to US Census data. Higher serum 25(OH)D was associated with lower rate of SARS-CoV-2 positivity (OR 0.98, 95% CI 0.98–0.99) in logistic regression models adjusted for age, sex, ethnicity, and latitude. Confounder adjustment in this study does not include important morbidities (e.g., obesity) that are known to strongly influence both vitamin D and COVID-19 risk. The confounders considered, such as ethnicity and latitude are crudely estimated and are prone to misclassification. Although this study provides insight from a large cohort, there is high risk of bias from residual confounding.

Larger, more fully characterised, cohorts have enabled more thorough consideration of confounding variables. Three studies have used data from the UK Biobank to consider the association baseline (2006–2010) measurements of serum 25(OH)D and incident COVID-19. In analysis of the first release of UK Biobank data (*n* = 265 cases), Hastie et al. [33] demonstrated negative univariable associations between 25(OH)D and COVID-19 status. However, consistent with the considerations described above, the association was attenuated to the null in multivariable models considering important confounders, including ethnicity, sex, month of 25(OH)D measurement, deprivation, body mass index (BMI), smoking, self-reported health measures, and comorbidities such as hypertension and diabetes. These findings were confirmed by Raisi-Estabragh et al. [34] in a larger data set of the UK Biobank cohort (*n* = 1326 cases), demonstrating that amongst participants tested for COVID-19, there was no statistically significant association between season-adjusted 25(OH)D levels and COVID-19 status in base models or with adjustment for age, sex, and ethnicity. Ma et al. [35] took a more extensive approach using the same data set, investigating the association of baseline serum 25(OH)D, genetically predicted vitamin D, and self-reported habitual vitamin D supplementation with COVID-19 test result in 8297 UK Biobank participants tested for COVID-19. Similar to Hastie et al. [33] and Raisi-Estabragh et al. [34] they found no association between serum 25(OH)D and COVID-19 status, nor with genetically predicted vitamin D levels. They reported a protective association with habitual vitamin D supplementation (OR 0.66; 95% CI 0.45–0.97). This is an interesting observation and the lack of consistency with serum and genetically determined vitamin D suggests that the observed protective effect from supplementation may be explained by confounding lifestyle and sociodemographic factors rather than vitamin D itself. However, a caveat with all three UK Biobank studies is that while they are prospective, which means that the COVID-19 cannot be responsible for the 25(OH)D measurement, the baseline 25(OH)D level was obtained approximately 10–14 years prior to COVID-19 testing. There is some evidence of tracking of 25(OH)D across life [29], but there is of course the potential for 25(OH)D status to change over that time interval, as can supplementation habits.

A further cohort study in Israel, including 7,807 children and adults with at least one prior blood test for 25(OH)D and subsequent COVID-19 testing (*n* = 782 positive) [36], documented possible associations between 25(OH)D levels and COVID-19 disease. In multivariable logistic regression models categorising 25(OH)D status into vitamin D levels below or above 30 ng/ml, 25(OH)D in the lower category was associated with significantly greater odds of COVID-19 positivity, and in a separate model with greater odds of hospitalisation. However, in this analysis, the lack of knowledge about the time interval between 25(OH)D measurement and COVID-19 testing as well as the observational nature of the study limits the inferences that can be drawn from the findings.

Several hospital-based studies provide further results. Meltzer et al. [37] conducted a review of hospital-based cases, identifying all patients tested for COVID-19 with a record of serum 25(OH)D measurement in the preceding 12 months. They categorised patients into vitamin D deficient, sufficient, and uncertain, based on 25(OH)D levels and subsequent prescription history. In fully adjusted models, they report higher odds of COVID-19 in the vitamin D deficient group, compared to the vitamin D sufficient cohort. Given the observational study design, the same caveats of potential residual or unmeasured confounding described previously, together with issues due to selection bias from the sampling based on routine 25(OH)D measurement, are relevant. Maghbooli et al. [38] studied the association of serum 25(OH)D with COVID-19 severity in 235 COVID-19 patients with 25(OH)D measured at the time of hospitalisation. They categorised vitamin D status into sufficient or deficient (< 30 ng/ml). In a backward logistic regression model after adjusting for age, sex, BMI, smoking, and history of a chronic medical disorder, there were independent associations between vitamin D sufficiency and decreased disease severity. In a similar study, Mendes et al. [39] investigated 160 older adults admitted to a geriatric ward with COVID-19 and serum 25(OH)D measured during the acute illness. Outcomes were poor with 25% in-hospital morality in the whole sample, which disproportionately affected men (63%). In sex-stratified multivariable Cox regression models they documented an independent association between lower vitamin D status and mortality in men, but not in women [40]. It is possible that the different relationship by sex reported by Mendes et al. reflects greater number of deaths in men than women, particularly as 25(OH)D levels were, in fact, higher in men. Luo et al. [41] present a single centre retrospective analysis from China, including 335 hospitalised COVID-19 patients and 560 age- and sex-matched controls. Serum 25(OH)D was measured at admission for cases and taken from within a defined 12 month period for the controls. Severity of COVID-19 was based on the level of respiratory involvement. In a general linear model adjusting for age, sex, BMI, and comorbidities, serum 25(OH)D was significantly lower among COVID-19 patient than in controls. In a multivariable logistic regression model, within the COVID-19 patients, vitamin D deficiency (< 30 nmol/l) was associated with greater COVID-19 severity (OR: 2.72; 95% CI: 1.23, 6.01, *p* < 0.05). In a study of 222 hospitalised patients screened for SARS-CoV-2 infection, by Alguwaihes et al. [42], vitamin D status was not associated with infection risk. In multivariable logistic regression models, severe 25(OH)D deficiency (< 12.5 nmol/l) was associated with increased mortality risk, although the association was not statistically significance [OR: 4.9; 95% CI: 0.9, 25.8; *p* = 0.06]. Importantly, in all of the aforementioned hospital-based studies, 25(OH)D was measured at time of COVID-19 hospitalisation, as vitamin D is an inverse acute phase reactant, with lower levels at times of physiological stress, these studies are at high risk of possible reverse causation. That is, that associations in these studies may be explained by greater levels of inflammation during acute illness reflecting more severe infection and causing a reduction in 25(OH)D, rather than the other way around. Such effects have been demonstrated in humans for surgical procedures and trauma [16], and in animal models for infection [17], but a small recent study documented no apparent effect in the setting of human malaria infection [43, 44]. Of course, the fact that acute inflammation may drive a reduction in 25(OH)D levels does not preclude the lower concentrations also having a biological effect, but at present the physiological significance of this inverse acute phase response remains to be elucidated.

Confounding and reverse causation, therefore, may be important drivers of relationships reported in observational studies. In an attempt to mitigate these sources of spurious association, Butler-Laporte et al. [45] used a two sample Mendelian Randomisation study design to investigate evidence of a causal link between genetically determined serum 25(OH)D and COVID-19 susceptibility. Genetic instruments for 25(OH)D levels were identified from meta-analysis of two genome-wide association studies comprising a total of 443,734 participants of European Ancestry. Genetic variants linked to COVID-19 susceptibility were obtained from six genome-wide association studies from four countries. The authors report no clear effect of genetically determined 25(OH)D on COVID-19 susceptibility. Importantly, however, the Mendelian Randomisation design tests the association between outcome and a lifelong genetic component of the exposure, with the genetic instrument explaining a very small proportion of the exposure variance, so necessitating very large cohorts to achieve adequate statistical power. Where the exposure is likely to be thresholded, such as vitamin D status, the actual level of the exposure in the population is likely critical [1, 4] and such studies should not be viewed as substitutes for properly conducted RCTs.

#### Interventional studies

At the time of writing, there are very limited data from interventional studies. Annweiler et al. [46] report a quasi-experimental study of 77 elderly patients consecutively hospitalised for COVID-19. The patients were categorised into three groups including individuals who had received regular bolus vitamin D supplementation in the preceding year as ascertained from primary care records (*n* = 29), those who received a single oral dose of 80,000 IU vitamin D3 within hours of COVID-19 diagnosis (*n* = 16), and those who received no vitamin D supplementation (*n* = 32). They considered associations with 14-day mortality and a COVID-19 disease severity score, adjusting for a wide range of confounders. The authors document less severe COVID-19 and lower mortality in individuals taking vitamin D supplementation in the preceding year, but not in those supplemented after COVID-19 diagnosis. Assessment of survival benefit at 14 days is likely too early for observation of an effect from increment of vitamin D levels as a result of supplementation at time of COVID-19 diagnosis. Furthermore, the authors do not measure serum vitamin D; therefore, associations described relate to recorded supplement use rather than directly measured vitamin D levels. In a similar study of 66 nursing home residents with COVID-19, Annweiller et al. [47] consider the relationship between vitamin D supplementation (80,000 IU vitamin D3 in the month preceding or the week following diagnosis of COVID-19) with COVID-19 severity score or mortality over mean follow-up time of 36 ± 17 days. The authors document association of bolus vitamin D3 supplementation during or just before COVID-19 with less severe COVID-19 and better survival rate. There was notable imbalance in sample size of the intervention (*n* = 57) and comparator groups (*n* = 9). Furthermore, the authors state that regular bolus supplementation (every 2–3 months) without measurement of serum vitamin D is recommended practice for nursing homes residents in the local setting (France). Given the absence of measured serum vitamin D, the high likelihood that all study participants were receiving regular supplementation (even if this was not administered within the selected window of this study), and the markedly imbalanced samples in the intervention and comparator groups, it is possible that the observed associations may be subject to statistical artefact.

Tan et al. [48] reported a quasi-experimental study exploiting a change in treatment protocols for COVID-19 patients in their hospital (Singapore General Hospital) from no supplementation to routine administration of a vitamin D, magnesium, and vitamin B12 combination supplement. Consecutive patients admitted after the change in policy were taken as the intervention group (*n* = 17), and those prior as comparators (*n* = 26). They included patients aged over 50 years hospitalised with COVID-19 and not requiring oxygen therapy or intensive care. They report significantly higher proportion of patients with clinical deterioration (oxygen therapy or intensive care) in the control (*n* = 16) than in the interventional group (*n* = 3). These results suggest a possible protective effect from supplementation. However, as a combined supplementation regimen was used, it is not possible to attribute any associations to a single agent. In addition, the interventional group comprised a cohort treated later on in the pandemic, it is likely that other aspects of their care may have improved, compared to the earlier patients used as controls, with increased experience of treating physicians with COVID-19. The small number of participants and events limits confounder adjustment in this analysis. On balance, there are multiple factors that preclude definitive attribution of the observed differences in this study to vitamin D supplementation.

Rastogi et al. [49] undertook a small randomised trial of the effect of high-dose short-term vitamin D supplementation on COVID-19 outcomes. They randomised 40 patients with polymerase chain reaction (PCR) confirmed SARS-CoV-2 to receive cholecalciferol 60,000 IU daily (*n* = 16) until achieving serum 25(OH)D > 50 ng/l (tested at day 7 and day 14), vs placebo (*n* = 24). PCR tests for SARS-CoV-2 were performed at 7-day intervals (7, 14, and 21 days). The authors report that, during the study period, 10 out of 16 (62.5%) participants in the intervention group achieved SARS-CoV-2 negativity compared to 5 out of 24 (20.8%) participants (*p* = 0.018) in the control arm. The mean duration to SARS-CoV-2 negativity was 17.6 ± 6.1 and 17.6 ± 6.4 days (*p* = 0.28) in the intervention and control arm, respectively. The randomisation method is not documented, and no reason is given for the unequal allocation to active and placebo groups. The intervention was not blinded, and analytical approach (“modified intention to treat”) is obscure. While the authors imply that a lower duration to SARS-CoV-2 negativity indicates more rapid viral clearance, data on severity of illness or time to discharge are not reported. All of these considerations, together with the very small size of this trial, substantially reduce any confidence in these findings.

Entrenas Castillo et al. [50] present pilot results from a RCT of the effect of calcifediol (25-hydroxy-vitamin D) supplementation on intensive care admission and death in a hospitalised cohort of 76 adults with PCR confirmed SARS-CoV-2 infection and radiographic evidence of viral pneumonia. There was “electronic” randomisation of consecutive eligible participants on admission at a ratio of 2:1 to intervention. Supplementation with calcifediol was undertaken on admission, day 3, day 7, and weekly thereafter at an initial dose of 0.532 mg and 0.266 mg for subsequent doses. The control group received standard medical care. There was no placebo pill for the controls. Calcifediol supplementation was stopped if a patient was admitted to intensive care. Of the 50 patients receiving calciferol, one (2%) required intensive care, compared to 50% (*n* = 13) in the control cohort (*p* < 0.001). There were no deaths in the intervention arm, compared with two deaths in the control group. These results suggest beneficial effects of calciferol supplementation in reducing severity of COVID-19 course. However, the results are much more dramatic than is biologically plausible, which raises the possibility that the observed effects are significantly inflated by systematic bias. Indeed, due to the small sample, despite randomisation, there is heterogeneous distribution of important morbidities such as hypertension and diabetes with greater rates of disease in the control group.

Murai et al. [51] undertook a more robustly conducted RCT from Brazil. They tested the effect of a single high dose of oral vitamin D (200,000 IU) on clinical outcomes of patients hospitalised with moderate–severe COVID-19 (PCR confirmed). Patients were randomly assigned to intervention (*n* = 120) or placebo (*n* = 120). Randomisation was allocated using a computer-generated code. Outcomes were defined a priori. The primary outcome was length of stay, a set of secondary in-hospital outcomes were also pre-specified. The authors reported no differences in length of stay, in-hospital mortality, admission to intensive care, or requirement for mechanical ventilation between the intervention and control groups. Contrary to the findings of Entrenas Castillo et al. [50] and Rastogi et al. [49], the findings from this larger and better conducted trial [51] do not support a role for vitamin D supplementation for treatment of moderate–severe COVID-19. However, given the still modest sample sizes, an effect is not excluded by these findings and further studies in larger populations with appropriately matched controls are needed, with randomised, blinded intervention designs as the optimal approach, albeit one which may be difficult to achieve given the practical considerations.

## Current guidance for vitamin D therapy in the context of COVID-19

In response to the discussion around the potential preventative and therapeutic role of vitamin D in COVID-19, public health agencies in the UK published three rapid evidence reviews in June 2020. A report conducted by the National Institute for Health and Care Excellence (NICE) and another by the Royal Society were specifically concerned with the role of vitamin D in the context of COVID-19 [52, 53]. A third report from the Scientific Advisory Committee on Nutrition (SACN) covered respiratory infections other than COVID-19 [54]. Furthermore, joint international guidance on vitamin D supplementation in the context of COVID-19 has also been published from the American Society for Bone and Mineral Research (ASBMR), Endocrine Society, American Association of Clinical Endocrinologists (AACE), European Calcified Tissue Society (ECTS), the National Osteoporosis Foundation (NOF), and the International Osteoporosis Foundation (IOF) [55]. These reports predominantly conclude that there is insufficient evidence to recommend use of vitamin D for treatment or prevention of respiratory tract infections or COVID-19 but endorse supplementation for maintenance of musculoskeletal health.

There is considerable variation between existing national guidelines, both in relation to recommended dietary intakes and minimum serum levels of vitamin D. The UK SACN report in 2016 used 25 nmol/l 25(OH)D as the threshold for defining the recommended nutrient intake (RNI) of 400 IU per day [56]. Groups at high risk of vitamin D insufficiency, and many individuals in the winter months, may require supplementation to achieve sufficient levels. At the time of writing, the UK government has issued free vitamin D supplements at 400 IU daily for care home residents and other vulnerable groups; while this dose is usually adequate to prevent severe deficiency in the context of population health, it may not provide reliable repletion in those at high risk of deficiency. However, it is important to note that SACN’s remit is risk assessment for deficiency of a nutrient at the population level, rather than optimum levels in individuals or recommendations for clinical treatment. Other guidelines suggest that higher doses, e.g., 800–3200 IU per day, may be required in this context to achieve repletion before settling on an appropriate lower maintenance dose [53, 54, 57, 58]. Indeed there have been recent calls for the UK RNI to be raised to 800 IU daily given the potential signals for benefit and absence of any safety concerns at this intake [59]. EU guidance suggests an intake of 600 IU daily and that from the US suggests 600 IU, or in those aged over 70 years, 800 IU daily [60–62]. Importantly, there is no evidence for additional benefit of pharmacological “mega-doses”; indeed such approaches have been associated with increased risk of falls and fractures [63], and of overt vitamin D toxicity [64].

## Ongoing and future work

The prevailing evidence is such that a benefit of vitamin D supplementation in the prevention or treatment of COVID-19 disease cannot be proved or refuted. The underlying biology suggests a role for vitamin D in modulating host responses to several respiratory pathogens [1, 65, 66], but whether these local cellular effects of 1,25(OH)_2_-vitamin D may be usefully influenced by vitamin D supplementation is uncertain. Thus, further work, including robustly designed and conducted RCTs, are needed. Queen Mary University of London has initiated a prospective national study, COVIDENCE UK, recruiting more than 18,000 participants [67]. In an initial online questionnaire information is being collected on determinants of vitamin D status and other important risk factors. These data are linked to notifications of incident COVID-19 identified from monthly online follow-up, cross-checked against routinely collected health outcome data held by NHS Digital. The “CORONAVIT” RCT, recruiting 6200 individuals, randomised in an open label design to either the UK recommendation of 400 IU/day (control), or 25(OH)D testing with replacement at a dose of 800 IU or 3200 IU/day is underway to investigate the potential for different vitamin D supplementation strategies to reduce the risk or severity of COVID-19 [68]. Several other trials are also underway or planned to investigate the preventative or therapeutic potential of vitamin D supplementation in the context of COVID-19 (Table 1).

**Table 1:** Selected ongoing and planned randomised controlled trials of the effect of vitamin D supplementation and COVID-19 outcomes in adults

Study title, country	Participant characteristics	*n*	Intervention	Primary outcome	Status
The effect of Vitamin D supplementation on COVID-19 recovery (COVID-VITD), Tunisia [70]	Asymptomatic and pauci-symptomatic COVID-19, within 14 days of positive RT-PCR	130	200,000 IU/1 ml of Cholecalciferol (1 ml), oral form	Time interval between the first positive RT-PCR and the second negative RT-PCR	Completed
Vitamin D and COVID-19 Trial (VIVID), United States of America [71]	Non-hospitalised individuals within 7 days of positive SARS-CoV-2 testing	2700	Daily vitamin D3 (9600 IU/day on days 1 and 2; 3200 IU/day on days 3 through 28)	Healthcare visits (including hospitalizations, emergency room visits, or ambulatory or virtual clinician visits) for symptoms or concerns related to COVID-19 or deaths in participants newly diagnosed with COVID-19 within 4weeks of diagnosis	Recruiting
Prevention of COVID-19 with oral vitamin D supplemental therapy in essential healthcare teams (PROTECT), Canada [72]	Health care workers caring for individuals at high risk of SARS-CoV-2 infection	2414	10 tablets containing 10,000 IU (total: 100,000 IU) of Vitamin D3 taken orally at baseline, followed by 10,000 IU weekly	Incidence of laboratory-confirmed COVID-19 infection over 16-week follow-up	Recruiting
Efficacy of Vitamin D supplementation to prevent the risk of acquiring COVID-19 in healthcare workers (COVID-19), Mexico [73]	Health care workers caring for patients with COVID-19	400	cholecalciferol 4,000 IU orally daily for 30 days	Incident COVID-19 confirmed by RT-PCR for SARS-CoV-2, or by antibody detection [Time frame: 45 days]	Recruiting
Trial of Vitamin D to reduce risk and severity of COVID-19 and other acute respiratory infections (CORONAVIT), UK [68]	UK residents ≥ 16 years old	6200	(1) Lower-dose vitamin D: offer of a daily dose of 800 IU cholecalciferol to individuals with 25-hydroxyvitamin D level < 75 nmol/l (2) Higher-dose vitamin D: offer of a daily dose of 3200 IU cholecalciferol to individuals with 25-hydroxyvitamin D level < 75 nmol/l	Proportion of participants experiencing at least one doctor-diagnosed or laboratory-confirmed acute respiratory infection of any cause	Recruiting
Cholecalciferol to improve the outcomes of COVID-19 patients (CARED), Argentina [74]	High risk patients hospitalised with confirmed SARS-CoV-2 infection, without respiratory compromise at baseline	1264	5 capsules containing 100,000 IU of vitamin D each. The intervention will be 5 capsules given in a one-time oral intake	Respiratory sequential organ failure assessment (SOFA) score. Need for high dose of oxygen or mechanical ventilation	Recruiting
Efficacy of Vitamin D treatment in mortality reduction due to COVID-19, Spain [75]	Hospitalised with RT-PCR positive SARS-CoV-2 infection and pneumonia and serum 25(OH)D < 30 mg/ml	108	If vitamin D deficiency (< 30 ng/ml) treatment with 2 capsules of 0.266 mg If vitamin D deficiency (< 40 ng/ml): treatment with 1 capsule of 0.266 mg	Mortality at 21 days	Recruiting
COVID-19 and Vitamin D supplementation: a multicenter randomized controlled trial of high dose versus standard dose vitamin D3 in high-risk COVID-19 Patients (CoVitTrial), France [76, 77]	Age ≥ 65 years, in hospital, nursing home, or outpatient setting with selected high risk features	260	Intervention: Patients receive a vitamin D supplementation of 400,000 IU in a single oral dose Control: patients receive a vitamin D supplementation of 50,000 IU in a single oral dose	All-cause death during the 14 days following the inclusion and intervention	Completed

*COVID-19* coronavirus disease 2019; *IU* international units; *RT-PCR* reverse transcriptase polymerase chain reaction; *SARS-CoV-2* severe acute respiratory syndrome coronavirus 2

## Conclusion

Although there are a growing number of studies into the links between vitamin D and COVID-19, they are mostly small observational studies with high risk of bias, residual confounding and reverse causality. There is need for adequately powered clinical trial data to more definitively investigate the role of vitamin D in COVID-19. At present, evidence to support vitamin D supplementation for the prevention or treatment of this infection is inconclusive. It seems likely, given shielding advice to individuals clinically vulnerable to severe COVID-19, that many frail elderly individuals will have experienced musculoskeletal deconditioning and reduced 25(OH)D status as a result of remaining indoors throughout the summer months (personal communication, UK Royal Osteoporosis Society). While the current evidence base does not support supplementation with vitamin D alone as a population health measure to prevent fracture [1, 69], it is clear that supplementation will reduce the risk of overt manifestations of vitamin D deficiency such as osteomalacia [1]. In this context, the supplemental strategy at the level of the population differs to that targeted to those overtly vitamin D deficient, but the evidence base clearly demonstrates that use of pharmacological mega-dose vitamin D is associated with no additional benefit but has potential for harm. Thus, in the absence of any further compelling data, adherence to current guidance on vitamin D supplementation (varying from 400 IU daily as the UK RNI up to 800 IU per day in the US population), usually predicated principally on maintaining musculoskeletal health, appears appropriate.
